# Risk Factors Associated with Outcomes of Recombinant Tissue Plasminogen Activator Therapy in Patients with Acute Ischemic Stroke

**DOI:** 10.3390/ijerph17020618

**Published:** 2020-01-18

**Authors:** Yi-Ju Tseng, Ru-Fang Hu, Shin-Tyng Lee, Yu-Li Lin, Chien-Lung Hsu, Shih-Wei Lin, Chia-Wei Liou, Jiann-Der Lee, Tsung-I Peng, Tsong-Hai Lee

**Affiliations:** 1 Department of Information Management, Chang Gung University, Taoyuan 33302, Taiwan; yjtseng@mail.cgu.edu.tw (Y.-J.T.);; 2 Department of Laboratory Medicine, Chang Gung Memorial Hospital at Linkou, Taoyuan 333, Taiwan; 3 Healthy Aging Research Center, Chang Gung University, Taoyuan 33302, Taiwan; 4 Department of Nursing, Chang Gung Memorial Hospital at Linkou, Taoyuan 333, Taiwan; 5 Department of Nursing, Chang Gung University of Science and Technology, Taoyuan 33302, Taiwan; 6 Graduate Institute of Business and Management, Chang Gung University, Taoyuan 33302, Taiwan; 7 Department of Visual Communication Design, Ming-Chi University of Technology, New Taipei City 24301, Taiwan; 8 Department of Nursing, Taoyuan Chang Gung Memorial Hospital, Taoyuan 333, Taiwan; 9 Department of Industrial Engineering and Management, Ming-Chi University of Technology, New Taipei City 24301, Taiwan; 10 Stroke Center and Department of Neurology, Linkou Chang Gung Memorial Hospital, Taoyuan 333, Taiwan; 11 Department of Neurology, Kaohsiung Chang Gung Memorial Hospital, Kaohsiung 83301, Taiwan; 12 College of Medicine, Chang Gung University, Taoyuan 33302, Taiwan; 13 Department of Neurology, Chiayi Chang Gung Memorial Hospital, Chiayi 613, Taiwan; 14 Department of Neurology, Keelung Chang Gung Memorial Hospital, Keelung 20401, Taiwan

**Keywords:** ischemic stroke, risk factor, thrombolytic therapy, rt-PA, outcome analysis

## Abstract

Ischemic stroke is the most common type of stroke, and early interventional treatment is associated with favorable outcomes. In the guidelines, thrombolytic therapy using recombinant tissue-type plasminogen activator (rt-PA) is recommended for eligible patients with acute ischemic stroke. However, the risk of hemorrhagic complications limits the use of rt-PA, and the risk factors for poor treatment outcomes need to be identified. To identify the risk factors associated with in-hospital poor outcomes in patients treated with rt-PA, we analyzed the electronic medical records of patients who were diagnosed with acute ischemic stroke and treated for rt-PA at Chang Gung Memorial Hospitals from 2006 to 2016. In-hospital death, intensive care unit (ICU) stay, or prolonged hospitalization were defined as unfavorable treatment outcomes. Medical history variables and laboratory test results were considered variables of interest to determine risk factors. Among 643 eligible patients, 537 (83.5%) and 106 (16.5%) patients had favorable and poor outcomes, respectively. In the multivariable analysis, risk factors associated with poor outcomes were female gender, higher stroke severity index (SSI), higher serum glucose levels, lower mean corpuscular hemoglobin concentration (MCHC), lower platelet counts, and anemia. The risk factors found in this research could help us study the treatment strategy for ischemic stroke.

## 1. Introduction

Ischemic stroke is the most common type of stroke, responsible for approximately 87% of all strokes worldwide [1], and it can cause loss of functions, such as speaking, moving, and reading. In Taiwan, according to statistics of the Ministry of Health and Welfare (MOHW), stroke fourth highest cause of death, and similar to the data published by the American Heart Association, approximately 70%–80% of stroke patients had ischemic stroke [2,3]. 

The guideline-recommended treatment for eligible patients with acute ischemic stroke is thrombolytic therapy using recombinant tissue-type plasminogen activator (rt-PA) to dissolve blood clots, which was approved by the U.S. Food and Drug Administration in 1996 for intravenous use within 3 h of stroke [4,5]. According to evidence, the guidelines completely describe the emergency evaluation and treatment with intravenous rt-PA therapies [6]. For instance, the guidelines recommend intravenous alteplase administration for selected patients who can be treated within 3 h or 3–4.5 h of ischemic stroke symptom onset. Physicians should review the criteria of use such as blood pressure of <185/110 mmHg and initial glucose levels of >50 mg/dL to determine patient eligibility [6]. The use of rt-PA is also limited by important contraindications, including coagulopathy, recent surgery, or stroke or head injury within the past 3 months [6].

Previous studies indicate that treatment with rt-PA may effectively improve neurological deficits in patients with acute ischemic stroke [7,8]. Early rt-PA treatment promotes independence and increases quality-adjusted life years (QALY) for stroke victims [9]. The prescription of rt-PA between 3 and 4.5 h after onset improves the clinical outcome [10] and the functional recovery [11] in patients with acute ischemic stroke. For long-term outcome evaluation, a study indicated that patients with acute ischemic stroke treated with rt-PA experience longer survival, delayed hospital readmission, and shorter time to independence [12]. Furthermore, patients at high risk of symptomatic intracranial hemorrhage still benefit from alteplase [13]. In addition to the time-to-treatment, stroke severity and stroke subtype might also affect the benefit from rt-PA treatment [14]. However, treatment with rt-PA requires careful consideration of both the risks and benefits, as bleeding is the most common side effect, which may cause the underuse of rt-PA despite guideline recommendations [15,16,17,18,19,20,21]. Despite the low use rate, one research concluded that larger hospitals were more likely to administer rt-PA [20]. Further efforts to improve appropriate administration of rtPA should be encouraged.

Previous studies used electronic medical records estimating risk factors associated with hemorrhage after rt-PA treatment. Patients diagnosed with hyperlipidemia, cardioembolism, severe stroke, or advanced age are at a higher risk of hemorrhaging, and the use of rt-PA should be considered carefully [22,23]. Under the condition that all patients are eligible for receiving rt-PA therapy based on the guidelines, only a few studies used both laboratory test results and medical history data to evaluate treatment outcomes [22,23,24]. However, using both laboratory test results and medical history data to identify the risk factors that lead to poor outcomes after rt-PA therapy in the general population still need to be explored [25].

For patients given rt-PA after stroke onset, we sought to investigate the general risk factors associated with poor rt-PA treatment outcomes. Therefore, our study carried out an analysis for patients in favorable and poor outcome groups to determine what factors might be associated with in-hospital poor outcomes, mainly in-hospital death, intensive care unit (ICU) stay, and prolonged length of hospital stay (LOS). 

## 2. Materials and Methods 

### 2.1. Data Source

This study was conducted using the pooled electronic medical records (EMRs) from the Stroke Registry of the Chang-Gung Healthcare System (SRICHS) [26] and the Chang Gung Research Database (CGRD) from the Chang Gung Memorial Hospitals (CGMHs), the largest group of healthcare providers in Taiwan. We analyzed the EMRs of patients who were diagnosed with acute ischemic stroke at CGMHs between 2006 and 2016, including 7 branches of CGMHs located in Linkou, Taipei, Taoyuan, Keelung, Yunlin, Chiayi, and Kaohsiung from northern to southern Taiwan. Medical histories defined by the history of diagnosis based on international classification of diseases (ICD) codes and laboratory test results were used as the variables of interest to determine the risk factors. The Chang Gung Medical Foundation Institutional Review Board approved this study (IRB no. 107-1113C) and granted waivers for patient consent.

### 2.2. Case Identification

This study included patients who had an emergency department visit for one of the top three diagnoses related to stroke (ICD-9-CM codes 433–436; ICD-10-CM codes I63, I65, I66, or I679) followed by hospitalization with a primary diagnosis of acute ischemic stroke (ICD-9-CM codes 433–434; ICD-10-CM codes I63) at CGMHs from 2006 to 2016 [27].

### 2.3. Outcome Definition

Three criteria were used in our study to define poor outcomes, including in-hospital death, intensive care unit (ICU) stay, and prolonged hospitalization. The in-hospital death is one of the worst outcomes of clinical care and has been evaluated in multiple studies [28,29,30]. ICU stay and prolonged hospitalization indicate that the patients need critical care or more time to recover after receiving rt-PA therapy [28,29,30,31]. In-hospital death was defined by death in hospital or a care home recorded on the discharge statement. Note that 24 patients without discharge statement records were considered as cases without in-hospital deaths. In CGMH, most of the patients receiving rt-PA therapy were admitted in the ICU for follow-up for 2 days. Therefore, all the patients treated with rt-PA will have at least 2 days of ICU stay. To identify the patients who need ICU admission owing to poor outcome, we set a threshold of 2 days to exclude the patients who were admitted in the ICU only for follow-up. The last criterion was prolonged hospitalization. The period between hospital admission and hospital discharge in an episode of care was calculated as the LOS. We defined prolonged hospitalization as the LOS of >55 days. The 55-day threshold was defined by the 90th percentile of the LOS in our data set [32]. If the time period between two hospitalizations, emergency department visits, and outpatient visits related to stroke was less than 3 days (i.e., a 3-day persistence window), these two visits were merged into a single episode of care, which is a means to apply consistent rules for medical conditions to infer distinct episodes of care, according to a previous study [33]. Patients fulfilling one of the criteria were included in the poor outcome group, but patients who fulfilled none of the criteria were included in the favorable outcome group.

### 2.4. Laboratory Assessments

Laboratory tests which were available in more than 80% of the ischemic stroke patients were included as a variable for further analysis: complete blood count (CBC), creatinine, sodium, alanine aminotransferase (ALT), blood glucose, and potassium levels. The glucose test can be performed using a traditional blood glucose test or finger stick blood glucose test. To include all the glucose test results, we collected both traditional and finger stick blood glucose test results to perform further analysis. Patients could have several laboratory tests during hospitalization. We only included laboratory tests that were collected within the stroke episode and those closest to the time before rt-PA treatment.

### 2.5. Medical History Assessment

To investigate medical histories, the Elixhauser comorbidity index developed by the Healthcare Cost and Utilization Project (HCUP) was applied to the diagnosis records [34]. We used the *emr* R package [35], a tool for integrating and processing EMRs, to evaluate the medical history variables for further analysis. This package can be used to group multiple ICD codes into a smaller number of clinically meaningful categories by the Elixhauser comorbidity classification. To exclude the medical histories that were recorded in only a few patients in our study population, we excluded the medical histories recorded in less than 5% of the patients in poor and favorable outcome groups. That is, we included the medical histories with more than 5% occurrence for the patients in poor or favorable outcome groups.

### 2.6. Stroke Severity Measurement

We use the stroke severity index (SSI) as a measure of stroke severity. The study by Sung et al. confirmed that the claims-based SSI is a valid substitute for the national institute of health stroke scale (NIHSS) score for estimating the stroke severity of patients hospitalized for acute ischemic stroke [36]. We calculated billing codes of Taiwan’s National Health Insurance for the SSI and separated it into three severities: mild (SSI ≤ 5), moderate (5 < SSI ≤ 12), and severe (SSI > 12) stroke according to previous studies [36,37].

### 2.7. Sensitivity Analyses

Two sensitivity analyses were applied to evaluate the effect of the proposed outcome definition, and the method we used to group the laboratory test results. The first sensitivity analysis defined a poor outcome as in-hospital death or ICU stay. Furthermore, instead of using real values of the test results, in the second sensitivity analysis, we categorized laboratory test results as normal, high, or low in accordance with the applicable reference ranges. This method can evaluate the effect of grouping laboratory test results by means of the reference range. Univariable and multivariable analyses were conducted in both sensitivity analyses.

### 2.8. Statistical Analysis

We performed a descriptive analysis of the characteristics of the patients in the favorable and poor outcome groups. Continuous variables were summarized as means (standard deviations) or medians (interquartile ranges) and discrete variables were summarized as frequencies and percentages. The means and medians were tested using Student’s t-test or the Kruskal–Wallis test, respectively. Chi-square tests or Fisher’s exact tests were used for the univariate analysis of the categorical variables. We performed least absolute shrinkage and selection operator (Lasso) regression in the multivariable analyses, which can address multicollinearity and is also an automated variable selection method [38]. The demographic characteristics, laboratory test results, and medical histories, which were different between the poor and favorable outcome groups (*p* value < 0.1), were kept as dependent variables in the Lasso model. The analyses were performed in R (version 3.4.4, The R Foundation for Statistical Computing, http://www.r-project.org/, R Core Team, Vienna, Austria). All statistical tests were two-sided, and statistical significance was defined as *p* < 0.05.

## 3. Results

### 3.1. Population Characteristics

**Figure 1:**
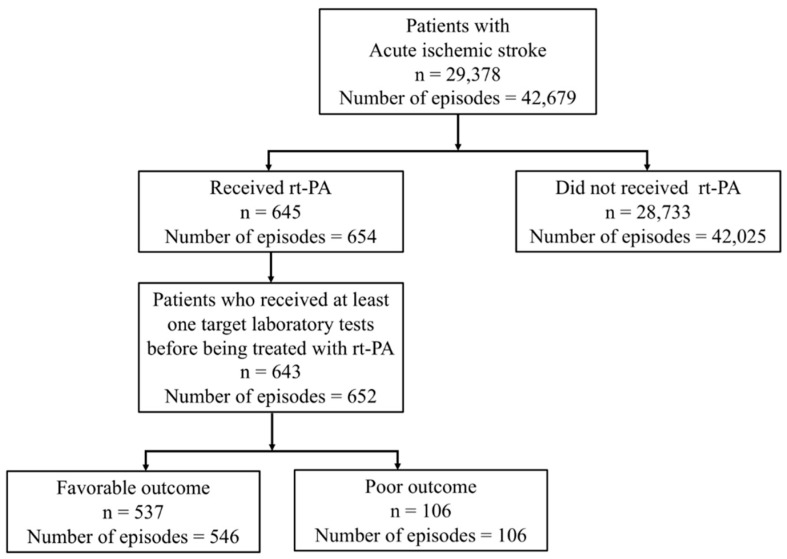
Study flow diagram. rtPA: recombinant tissue-type plasminogen activator.

In total, 42,679 episodes of care from 29,378 patients were eligible for the study. Approximately 2% of patients (645 individuals) received rt-PA after acute ischemic stroke onset. Among them, at least one targeted laboratory test was given in 652 episodes of care (643 patients) before rt-PA (Figure 1). As shown in Figure 1, among the patients who were diagnosed with acute ischemic stroke and given rt-PA, 537 had favorable outcomes and 106 had poor outcomes. Table 1 describes the demographic characteristics and stroke severity of these patients. Compared with patients with favorable outcomes, patients with poor outcomes were more likely to be older and have higher SSIs. The standardized residuals of stroke severity are shown in Table S1. The distribution of sex was also different between the two groups. Among the laboratory test results, hemoglobin level, mean corpuscular hemoglobin concentration (MCHC), platelet counts, and glucose levels were different between patients with favorable and poor outcomes (Table 2). Higher glucose values, lower hemoglobin levels, lower MCHCs, and lower platelet counts were significantly associated with an increased risk of a poor outcome.

### 3.2. Medical History Analysis

The medical histories of the patients, defined based on the Elixhauser comorbidity classification, are summarized in Table 3. The two groups did not differ in terms of their medical histories (all *p*-values  >  0.05).

### 3.3. Multivariable Analysis

We included laboratory test results and medical histories that were different (*p*-value < 0.1) between patients with favorable and poor outcomes and their demographic characteristics as independent variables in the Lasso model. Table 4 shows the model’s coefficients (lambda = 0.009326033). Only the important variables whose coefficient was not zero are shown. Variables that emerged as risk factors of poor treatment outcome for patients with acute ischemic stroke included patients who were female and had anemia, a more severe SSI, a higher glucose value, lower MCHC, and a lower platelet count.

### 3.4. Sensitivity Analysis: Outcome Definition

In the sensitivity analysis, we redefined a poor outcome as in-hospital death or ICU stay. There were 591 and 61 episodes in the new favorable and poor outcome groups, respectively. Compared with the univariate analysis of patient characteristics, the variables that were significantly different between the two groups were the same in this sensitivity analysis (Table S2). In the univariate analysis of the laboratory test results, higher glucose and potassium values were significantly associated with an increased risk of poor treatment outcome (Table S3). In contrast to the primary analysis (Table 2), the platelet counts not different, but the RBC counts and potassium levels were different between the two groups in this sensitivity analysis. The results of the medical histories are described in Table S4; only hypertension complicated by other diseases was associated with poor outcomes. The risk factor selected by Lasso in this analysis was SSI, whose coefficient was 0.146 and lambda was 0.1519911.

### 3.5. Sensitivity Analysis: Transformed Laboratory Test Results

In the second sensitivity analysis, we divided the laboratory test results into categories based on their reference ranges, and the univariate analysis results are shown in Table S5. This table shows that poor outcomes are associated with abnormal levels of hemoglobin, hematocrit, RBCs, and glucose. Above normal values of hemoglobin and glucose were more likely to result in a poor prognosis. Regarding risk factors, after transforming the laboratory test results, gender, SSI, hemoglobin, hematocrit, sodium, glucose, and a history of anemia were considered relevant factors selected by the Lasso method, as shown in Table S6. Similar to the primary analysis results, being female, having a higher SSI and glucose level, and anemia, were also found to be important features in this sensitivity analysis.

## 4. Discussion

Due to undesirable side effects, such as hemorrhaging, and low usage rates of rt-PA [39], it is essential to identify risk factors relative to treatment outcomes. We included demographic information, medical histories, and laboratory test results to determine the variables associated with the outcome of rt-PA therapy. Female patients, patients with anemia, patients with a higher SSI and glucose level, and lower MCHCs and platelet counts were more likely to have poor outcomes after receiving rt-PA. Stroke severity was selected as an important risk factor in the primary analysis and in the two sensitivity analyses. Anemia, female gender, and higher serum glucose levels were selected in the primary analysis and the sensitivity analysis, which grouped laboratory results based on their reference ranges. In the sensitivity analysis that used an alternative outcome definition, only SSI was kept in the Lasso model. The possible reason is that the number of episodes in the alternative poor outcome groups was only 61; the alternative poor outcome group was much smaller than the favorable outcome group.

Previous studies have shown that stroke severity and serum glucose level had a significant influence on intracerebral hemorrhage after intravenous tissue plasminogen activator therapy [22,23,24,40], which was consistent with our findings, even though we used SSI as a substitute for NIHSS score as a measure of stroke severity. In addition to the glucose level, diabetes may be associated with poor outcome of rt-PA therapy [41,42]. One study found that in the diabetic subgroup, the glycated hemoglobin index was positively correlated with symptomatic intracranial hemorrhage [40]. Instead of focusing on a specific subgroup, we considered all the patients with acute ischemic stroke who were treated with rt-PA as study cases. In the general population, the glycated hemoglobin index test was performed in only a few patients; therefore, we only included the glucose level in laboratory assessments. A recent study used functional ambulatory status as an outcome to investigate the association between risk factors and outcomes in ischemic stroke patients who received rt-PA and were on antihypertensive medications [24]. In our study, we defined poor outcomes as in-hospital death, ICU stay, and prolonged LOS, which are different from the published study.

Gender differences among patients with acute ischemic stroke who received rt-PA have been discussed in many studies [43,44,45,46,47,48,49,50,51]. A systematic review suggested that no gender difference existed in the outcome among patients treated with intravenous rt-PA [45]. The clinical outcome and the number of patients with a favorable outcome did not differ between women and men [50]. Few studies have reported that the usual gender difference in outcomes favoring men was not observed among patients treated with rt-PA [43,44,47,51]. However, similar to the findings of our study, few studies showed that women have a higher poststroke mortality, rate of disability, depression, and dementia, and poorer mRS (modified rankin scale) scores at discharge compared with men [48,49]. Gender differences in symptoms at presentation may create treatment delays for women [52]. The difference found in this study could possibly be explained by physiologic derangement not included in the dataset [14,50]. Similar to a previous study [46], we found that middle-aged women have a better outcome than middle-aged men, whereas at a more advanced age, men have a better outcome than women. However, the differences are not statistically significant. The gender differences among rt-PA therapy are still different across studies, and further research is required.

Our study used the Lasso approach to select significant features in the multivariable analysis because there was a high correlation between MCHC, hemoglobin levels, and hematocrit levels. In addition, some studies reported that the regression coefficients in a stepwise selection model may have considerable bias [53,54]. Similarly, with stepwise regression, Lasso, which adds regularization to penalize the number of parameters in the model, prevents overfitting and prevents multicollinearity.

The main feature of our study is that we considered the demographic characteristics, laboratory test results, and medical history for exploring the risk factors for poor outcomes after receiving rt-PA. Only a few studies include laboratory test results for rt-PA treatment outcome analysis and no study has investigated the associations between laboratory results and poor treatment outcome in the general population of patients with acute ischemic stroke; we found that glucose level, MCHCs, and platelet counts were associated with the treatment outcome. Moreover, because laboratory test results can be described as normal and abnormal, and the definition of treatment outcome can affect the analysis results, we also evaluated the effect of changing the definition of the variables. To address the problems mentioned above, we conducted sensitivity analyses to evaluate the effect of applying different outcome definitions and grouping the laboratory test results based on their reference ranges. Our results can provide prognostic information about using rt-PA for ischemic stroke.

Our study has several limitations. First, the number of patients who were treated with rt-PA was only 643, due to the low usage of rt-PA, although we included 11 years of data and 42,679 acute ischemic stroke episodes. Second, Charlson comorbidities have often been used to measure comorbidities [55]. However, the Charlson comorbidity index only includes 17 comorbidity categories, and some important diseases related to rt-PA outcome evaluation, such as anemia, are not included in the Charlson comorbidity index. To extensively define and analyze the medical histories of stroke patients, we chose Elixhauser comorbidities index, which includes 30 comorbidity categories, to group the diagnoses [34,56]. Third, medical histories might be missing if patients do not have a related diagnosis in their EMR. However, in our study group, more than 70% of the patients had visited CGMH before having a stroke. Moreover, in-hospital EMRs alone should only be used to build a risk model in worst-case scenarios wherein unconscious patients are admitted to the emergency department and no additional information can be provided. Fourth, patients included in the study might be ineligible for rt-PA treatment based on the guidelines. In our study, we included all patients who were given rt-PA for ischemic stroke to ensure a sufficient number of cases and to reflect the conditions in the real world. Another limitation is that time-to-treatment data, which is known as a factor associated with outcomes, was not available in our dataset. A previous study concluded that thrombolytic therapy beyond the 4.5 h time window seems to be associated with a significant increase in mortality in clinical practice [25]. However, according to previous studies, the third quartile of onset-to-treatment time was less than 3 h in Taiwan [2,57], that is, only a small proportion of cases was treated beyond the 4.5 h time window. The other clinical features that are important in predicting the clinical outcome, such as the subtype of stroke [46], cerebral arterial recanalization [58], and the presence and site of occlusion [58] could not be extracted from our dataset. These data should be collected for further analysis, including for building predictive models. For the NIHSS value at admission [46], we used the SSI, a valid substitute for the NIHSS score [36], as a measure of stroke severity. 

## 5. Conclusions

We analyzed the risk factors that may be associated with unfavorable rt-PA treatment outcomes. Our findings demonstrated that female gender, higher serum glucose levels, lower MCHC, lower platelet counts, history of anemia, and severe stroke were the risk factors relevant to rt-PA treatment outcomes, and these findings could help us study the treatment strategy for acute-stage ischemic stroke.

## Figures and Tables

**Table 1:** Univariate analysis of the patient characteristics.

Patient Characteristics	Favorable Outcome (*n* = 546)	Poor Outcome (*n* = 106)	*p* Value
Sex (%)			0.013 *
Male	356 (65.2)	55 (51.9)	
Female	190 (34.8)	51 (48.1)	
Age (median [IQR ^a^, Q1–Q3 ^b^])	66.00 [18, 56.00–74.00]	71.00 [18.75, 59.25–78.00]	0.001 *
SSI ^c^ (median [IQR, Q1–Q3])	9.65 [3.51, 8.28–11.79]	15.21 [11.34, 10.20–21.54]	<0.001 ***
Stroke severity (%)			<0.001 ***
Mild	99 (18.1)	14 (13.2)	
Moderate	313 (57.3)	20 (18.9)	
Severe	134 (24.5)	72 (67.9)	

* *p* < 0.05, *** *p* < 0.001, ^a^ IQR, interquartile range [first quartile, third quartile], ^b^ Q1–Q3, quartile 1–3, ^c^ Stroke severity index. SSI: stroke severity index.

**Table 2:** Univariate analysis of the laboratory results.

Laboratory Tests	Favorable Outcome (*n* = 546)	Poor Outcome (*n* = 106)	*p* Value
Creatinine (median [IQR ^a^, Q1–Q3 ^b^])	0.94 [0.36, 0.78–1.14]	0.95 [0.44, 0.79–1.23]	0.472
Hemoglobin (median [IQR, Q1–Q3])	14.20 [2.3, 13.00–15.30]	13.70 [2.6, 12.30–14.90]	0.015 *
Hematocrit (median [IQR, Q1–Q3])	41.70 [5.8, 38.70–44.50]	41.10 [6.2, 37.30–43.50]	0.053
MCH (mean corpuscular hemoglobin) (median [IQR, Q1–Q3])	30.50 [2.4, 29.30–31.70]	30.60 [2.4, 29.30–31.70]	0.826
MCHC (mean corpuscular hemoglobin concentration) (median [IQR, Q1–Q3])	33.90 [1.5, 33.20–34.70]	33.60 [1.8, 32.80–34.60]	0.024 *
MCV (mean corpuscular volume) (median [IQR, Q1–Q3])	89.60 [6, 86.50–92.50]	90.20 [6.2, 86.90–93.10]	0.259
Sodium (median [IQR, Q1–Q3])	139.00 [3.85, 137.15–141.00]	138.90 [3, 137.00–140.00]	0.116
Platelets (median [IQR, Q1–Q3])	206.00 [72, 170.00–242.00]	186.00 [64, 159.00–223.00]	0.007 **
RBCs (red blood cells) (median [IQR, Q1–Q3])	4.69 [0.67, 4.36–5.03]	4.69 [0.85, 4.15–5.00]	0.116
RDW (red cell distribution width) (median [IQR, Q1–Q3])	13.60 [3.6, 12.90–16.50]	13.60 [2.5, 12.80–15.30]	0.608
WBCs (white blood cells) (median [IQR, Q1–Q3])	7.90 [3.5, 6.40–9.90]	7.50 [3, 6.50–9.50]	0.639
ALT (alanine aminotransferase) (median [IQR, Q1–Q3])	23.00 [15, 17.00–32.00]	22.50 [17, 16.00–33.00]	0.694
Glucose (median [IQR, Q1–Q3])	128.00 [47, 110.00–157.00]	144.00 [56, 124.00–180.00]	<0.001 ***
Potassium (median [IQR, Q1–Q3])	3.70 [0.5, 3.44–3.94]	3.70 [0.42, 3.57–3.99]	0.139

* *p* < 0.05, ** *p* < 0.01, *** *p* < 0.001, ^a^ IQR, interquartile range [first quartile, third quartile], ^b^ Q1–Q3, quartile 1–3.

**Table 3:** Univariate analysis of the medical history variables.

Medical Histories (%)	Favorable Outcome (*n* = 546)	Poor Outcome (*n* = 106)	*p*-Value
Deficiency anemias	11 (2.0)	6 (5.7)	0.068
Congestive heart failure	42 (7.7)	7 (6.6)	0.851
Diabetes without chronic complications	85 (15.6)	16 (15.1)	1
Hypertension, uncomplicated	187 (34.2)	33 (31.1)	0.611
Hypertension, complicated	21 (3.8)	6 (5.7)	0.554
Liver disease	28 (5.1)	8 (7.5)	0.444
Chronic pulmonary disease	45 (8.2)	12 (11.3)	0.401
Solid tumor without metastasis	29 (5.3)	8 (7.5)	0.496
Valvular disease	34 (6.2)	8 (7.5)	0.771

**Table 4:** Risk factors identified by the Lasso model and their associated coefficients.

Selected Variable	Coefficient
Anemia	0.752
Sex: Male	−0.178
SSI ^a^	0.887
MCHC	−0.042
Platelet Count	−0.142
Glucose	0.200

^a^ Stroke severity index

## Supplementary Materials

The following are available online at https://www.mdpi.com/1660-4601/17/2/618/s1, Table S1. The standardized residuals of stroke severity, Table S2. Univariate analysis of the patients in the sensitivity analysis–outcome definition. Table S3. Univariate analysis of the laboratory results in the sensitivity analysis–outcome definition. Table S4. Univariate analysis of the medical history variables in the sensitivity analysis–outcome definition. Table S5. Univariate analysis of laboratory categorical variables in the sensitivity analysis–transformed laboratory test results. Table S6. Risk factors identified by the Lasso model (lambda: 0.01072267) and their associated coefficients in the sensitivity analyses–transformed laboratory test results.
