# Supplementary material for: Risk Factors Associated with Outcomes of Recombinant Tissue Plasminogen Activator Therapy in Patients with Acute Ischemic Stroke
Source: Int J Environ Res Public Health. 2020 Jan 18;17(2):618. doi: 10.3390/ijerph17020618 (PMC7014350; doi:10.3390/ijerph17020618)
Supplement: Supplementary file 1 [file ijerph-17-00618-s001.pdf]

Supplementary Materials

Table S1. The standardized residuals of stroke severity.

Stroke Severity	Favorable Outcome (n = 546)	Poor Outcome (n = 106)
Mild	1.226	−1.226
Moderate	7.248	−7.248
Severe	−8.792	8.792

Table S2. Univariate analysis of the patients in the sensitivity analysis–outcome definition.

Patient Characteristics	Favorable Outcome (n = 591)	Poor Outcome (n = 61)	*p* Value
Sex (%)			0.013 *
Male	382 (64.6)	29 (47.5)	
Female	209 (35.4)	32 (52.5)	
Age (median [IQR [a], Q1–Q3 [b]])	66.00 [18, 56.00–74.00]	72.00 [15, 63.00–78.00]	0.001 *
SSI [c] (median [IQR, Q1–Q3])	9.65 [3.51, 8.28–11.79]	21.17 [5.28, 18.03–23.31]	<0.001 ***
Stroke severity (%)			<0.001 ***
Mild	113 (19.1)	0 (0.0)	
Moderate	331 (56.0)	2 (3.3)	
Severe	147 (24.9)	59 (96.7)	

* $p < 0.05$, *** $p < 0.001$, [a] IQR, interquartile range [first quartile, third quartile], [b] Q1–Q3, quartile 1–3, [c] Stroke severity index.

Table S3. Univariate analysis of the laboratory results in the sensitivity analysis–outcome definition.

Laboratory Tests	Favorable Outcome (n = 591)	Poor Outcome (n = 61)	*p*-Value
Creatinine (median [IQR [a], Q1–Q3 [b]])	0.93 [0.36, 0.78–1.14]	1.01 [0.45, 0.80–1.25]	0.123
Hemoglobin (median [IQR, Q1–Q3])	14.10 [2.3, 13.00–15.30]	13.60 [2.3, 12.30–14.60]	0.011 *
Hematocrit (median [IQR, Q1–Q3])	41.70 [5.8, 38.70–44.50]	40.70 [6.9, 36.60–43.50]	0.053
MCH (mean corpuscular hemoglobin) (median [IQR, Q1–Q3])	30.50 [2.4, 29.30–31.70]	30.60 [2.8, 29.00–31.80]	0.892
MCHC (mean corpuscular hemoglobin concentration) (median [IQR, Q1–Q3])	33.90 [1.5, 33.20–34.70]	33.40 [1.9, 32.60–34.50]	0.019 *
MCV (mean corpuscular volume) (median [IQR, Q1–Q3])	89.60 [5.9, 86.60–92.50]	90.80 [6.4, 86.70–93.10]	0.262
Sodium (median [IQR, Q1–Q3])	139.00 [3.8, 137.20–141.00]	138.60 [3, 137.00–140.00]	0.105
Platelets (median [IQR, Q1–Q3])	205.00 [69, 169.00–238.00]	193.00 [69, 165.00–234.00]	0.248
RBCs (red blood cells) (median [IQR, Q1–Q3])	4.70 [0.67, 4.36–5.03]	4.55 [0.88, 4.09–4.97]	0.035 *
RDW (red cell distribution width) (median [IQR, Q1–Q3])	13.50 [3, 12.90–15.90]	14.20 [7, 12.90–19.90]	0.151
WBCs (white blood cells) (median [IQR, Q1–Q3])	7.80 [3.5, 6.40–9.90]	7.70 [2.9, 6.60–9.50]	0.815
ALT (alanine aminotransferase) (median [IQR, Q1–Q3])	23.00 [15, 17.00–32.00]	22.00 [14, 16.00–30.00]	0.329
Glucose (median [IQR, Q1–Q3])	130.00 [49, 111.00–160.00]	145.00 [55.5, 122.00–177.50]	0.006 **
Potassium (median [IQR, Q1–Q3])	3.70 [0.49, 3.44–3.93]	3.83 [0.5, 3.60–4.10]	0.003 **

* $p < 0.05$, ** $p < 0.01$, [a] IQR, interquartile range, [b] Q1–Q3, quartile 1–3.

Table S4. Univariate analysis of the medical history variables in the sensitivity analysis–outcome definition.

Medical Histories (%)	Favorable Outcome (n = 591)	Poor Outcome (n = 61)	*p* Value
Deficiency anemias	13 (2.2)	4 (6.6)	0.107
Congestive heart failure	42 (7.1)	7 (11.5)	0.328
Diabetes without chronic complications	91 (15.4)	10 (16.4)	0.985
Hypertension, uncomplicated	198 (33.5)	22 (36.1)	0.794
Hypertension, complicated	21 (3.6)	6 (9.8)	0.045 *
Liver disease	33 (5.6)	3 (4.9)	1
Chronic pulmonary disease	52 (8.8)	5 (8.2)	1
Solid tumor without metastasis	30 (5.1)	7 (11.5)	0.077
Valvular disease	36 (6.1)	6 (9.8)	0.39

* $p < 0.05$.

Table S5. Univariate analysis of laboratory categorical variables in the sensitivity analysis–transformed laboratory test results.

Laboratory Tests	Favorable Outcome (n = 546)	Poor Outcome (n = 106)	*p* Value
Creatinine (%)			**0.06**
High	96 (17.8)	29 (27.9)	
Low	7 (1.3)	1 (1.0)	
Normal	436 (80.9)	74 (71.2)	
Hemoglobin (%)			**0.015 ***
High	15 (2.8)	6 (5.7)	
Low	117 (21.5)	33 (31.4)	
Normal	413 (75.8)	66 (62.9)	
Hematocrit (%)			**0.011 ***
High	7 (1.3)	6 (5.7)	
Low	148 (27.2)	33 (31.4)	
Normal	390 (71.6)	66 (62.9)	
MCH (mean corpuscular hemoglobin) (%)			**0.482**
High	15 (2.8)	3 (2.9)	
Low	40 (7.3)	11 (10.5)	
Normal	490 (89.9)	91 (86.7)	
MCHC (mean corpuscular hemoglobin concentration) (%)			**0.837**
High	4 (0.7)	0 (0.0)	
Low	8 (1.5)	2 (1.9)	
Normal	533 (97.8)	103 (98.1)	
MCV (mean corpuscular volume) (%)			**0.366**
High	14 (2.6)	5 (4.8)	
Low	41 (7.5)	9 (8.6)	
Normal	490 (89.9)	91 (86.7)	
Sodium (%)			**0.005 ****
Low	13 (2.4)	9 (8.5)	
Normal	531 (97.6)	97 (91.5)	
Platelets (%)			**0.121**
High	7 (1.3)	2 (1.9)	
Low	63 (11.6)	19 (18.1)	
Normal	475 (87.2)	84 (80.0)	
RBCs (red blood cells) (%)			**0.013 ***
High	14 (2.6)	7 (6.7)	
Low	80 (14.7)	23 (21.9)	
Normal	451 (82.8)	75 (71.4)	

RDW (red cell distribution width) (%)			1
High	184 (33.8)	35 (33.3)	
Normal	361 (66.2)	70 (66.7)	
WBCs (white blood cells) (%)			**0.299**
High	108 (19.8)	19 (18.1)	
Low	3 (0.6)	2 (1.9)	
Normal	434 (79.6)	84 (80.0)	
ALT (Alanine aminotransferase) (%)			0.386
High	95 (17.9)	23 (22.1)	
Normal	435 (82.1)	81 (77.9)	
Glucose (%)			**0.01 ***
High	188 (37.2)	49 (52.7)	
Low	1 (0.2)	0 (0.0)	
Normal	317 (62.6)	44 (47.3)	
Potassium (%)			**0.128**
High	4 (0.7)	1 (1.0)	
Low	191 (35.3)	27 (26.0)	
Normal	346 (64.0)	76 (73.1)	

* $p < 0.05$, ** $p < 0.01$ Note. The p value printed in bold uses Fisher's exact test; otherwise, the chi-square test.

Table S6. Risk factors identified by the Lasso model (lambda: 0.01072267) and their associated coefficients in the sensitivity analyses–transformed laboratory test results.

Selected Variables	Coefficient
Anemia	0.776
Sex: Male	−0.205
SSI	0.828
Hemoglobin: High	0.954
Hemoglobin: Low	0.002
Hematocrit: High	0.377
Sodium: Low	0.886
Glucose: High	0.306
